# Data-Driven Monitoring of Probiotic Fermentation in Fruit Juices Using Near-Infrared Spectroscopy and Aquaphotomics: An Innovative Approach to Food Valorization

**DOI:** 10.3390/foods14071274

**Published:** 2025-04-05

**Authors:** Lueji Regatieri, Flora Vitalis, Erika Bujna, Quang Duc Nguyen, Zoltan Kovacs

**Affiliations:** 1 Department of Measurement and Process Control, Institute of Food Science and Technology, Hungarian University of Agriculture and Life Sciences (MATE), 1118 Budapest, Hungary; regatieri.santos.lueji@phd.uni-mate.hu (L.R.); vitalis.flora@uni-mate.hu (F.V.); 2 Department of Bioengineering and Alcoholic Drink Technology, Institute of Food Science and Technology, Hungarian University of Agriculture and Life Sciences (MATE), 1118 Budapest, Hungary; bujna.erika@uni-mate.hu (E.B.); nguyen.duc.quang@uni-mate.hu (Q.D.N.)

**Keywords:** fruit juice, probiotic bacteria, probiotic drink, near-infrared spectroscopy, chemometrics, aquaphotomics

## Abstract

The nutritional effects of fruit juices, combined with the added value of a probiotic, provide a plant-based fortified functional food. Some process-related drawbacks are caused by the pH parameter, which will affect the survival of probiotics during their industrial processing and storage. By means of developing a monitoring method for probiotic activity, the present study aims to investigate the application of near-infrared spectroscopy (NIR) as a correlative analytical method for fermentation process tracking, in association with the different absorption patterns of bound water, explained by aquaphotomics. The data evaluated in the wavelength range of 1300–1600 nm indicate classification accuracies of 99–100% and 99–93% during calibration and validation, respectively, when applying PCA-LDA for discriminating the fermentation times, for each one of the single and mixed bacterial groups. During PLSR prediction, according to the fermentation times, the validation models developed for pH show coefficients of determination in the range of 0.96 to nearly 1 and root mean square errors of 0.05 and 0.19. On the other hand, for the PLSR prediction of log cell count (CFU/mL), validation modeling shows a coefficient of determination of 0.85 and a root mean square error of 0.23. All things considered, the results support the applicability of combining NIR and aquaphotomics as a bioprocess monitoring tool, which can be further implemented in different studies and industrial contexts.

## 1. Introduction

The growing interest of consumers in adopting a healthier lifestyle through food and dietary supplements has enhanced the role of a balanced diet and the interest of the scientific community in conducting studies related to the performance of foods in reducing the risk of certain diseases, such as insulin resistance, diabetes, lactose intolerance, gastrointestinal disorders (irritable bowel syndrome, diarrhea), allergies, and obesity. The focus of the current research is on new natural ingredients, and the development of new functional foods is continuously increasing, using evidence-based approaches to improve diets and lifestyles, especially regarding probiotics [1].

Due to the growing popularity of probiotic foods, a consultation organized by the World Health Organization (WHO) and the Food and Agriculture Organization (FAO) proposed the following definition of probiotics in 2001, updated later in 2014: “live microorganisms that, when administered in adequate amounts, confer a health benefit on the host”. Furthermore, to be consumed, probiotics must be safe for their intended use—which requires clinical trials and regulatory approval—and they must have appropriate viability regarding the cell count at the end of shelf life and proven health benefits [2].

The most widely used probiotic bacteria are those of the lactic acid bacteria (LAB) group and their genera, including *Lactobacillus*, *Lactococcus*, *Streptococcus*, *Enterococcus*, and the genera *Bifidobacterium*. As previously mentioned, a certain population is required for the intended promotion of health beneficial effects, intrinsically related to LAB metabolic features. This type of bacteria can decompose macromolecular substances in food, such as polysaccharides and proteins—for example, through the degradation of casein, reducing the allergenicity of dairy products—in addition to producing acids or aromatic compounds and having the ability to inhibit certain types of bacteria. Based on these characteristics, a major fermentation strategy is to produce lactic acid during carbohydrate fermentation, which is species- and strain-dependent, presenting some limitations related to nutritional requirements and their impact on metabolic activities. Not only should the LAB strains be carefully selected for particular food matrices, but also, an optimal adjustment of physico-chemical conditions should be taken into consideration to ensure their viability [3,4,5,6,7]. According to their hexose metabolic pathway, LAB strains can be classified into (i) homofermentative, in which the conversion of the carbohydrate results mainly in lactic acid, and (ii) heterofermentative, where other end-products, such as acetic acid, carbon dioxide, or volatile acids, are also produced [8].

Probiotics have been mainly consumed in fermented dairy products (mainly milk and yogurt); however, due to current dietary restrictions and trends, alternative products are being developed or adapted to fulfill special requirements, such as lower levels of cholesterol, lactose free, dairy free, vegetarian, and vegan. The new market niches came along with plant-based probiotic products, through which a variety of fruit juices has been further studied [7] as ideal substrates for probiotics, since they contain all the macro- and micronutrients, such as carbohydrates, vitamins, minerals, polyphenols, and flavonoids. Stone fruits, in particular cherries and plums, could play a prominent role in the future production of probiotic products due to their essential composition. These fruits are a rich source of anthocyanins, flavanols, flavan-3-ols, phenolic acids, polysaccharides, alkaloids, and chlorophyll catabolite [9,10,11]. Sour cherry also has high anthocyanin content, bringing the beneficial aspects of anthocyanin: improvement in cardiovascular health, reduction in thrombogenesis, anti-diabetic effects, and anti-inflammatory effects, and prevention of neurodegenerative diseases [12].

Probiotic fruit juice quality and monitoring assessment typically includes pH, acidity, metabolite content, and viscosity measurements. These also involve determining phenolic content, antioxidant capacity, total sugars, organic acids, and ethanol concentration, in addition to microbiological analyses such as microbial counts, gram staining, and catalase tests. All these traditional instrumental reference tests are costly and time-consuming. Consequently, they do not allow for real-time monitoring and data-driven control of the fermentation process.

The advantage of using an indirect and non-destructive method via vibrational spectroscopy (near-infrared (NIR), mid-infrared, and Raman spectroscopy) is that it provides real-time analysis, used in combination with various chemometric methods, enabling its use for supervised classification or the development of prediction models. One feature of these may be that the otherwise significantly correlated NIR data are compressed into a smaller number of variables that are no longer correlated. The most commonly used chemometric methods are, e.g., principal component analysis (PCA), linear discriminant analysis (LDA), support vector machine (SVM), multiple regression analysis (MLR), and partial least squares regression (PLSR). Furthermore, spectroscopic techniques offer several benefits, such as portability for in situ applications, cost reduction, ease of operation, and the reliability of non-destructive methods, thus allowing real-time data-driven control of the fermentation process [13,14,15]. Recently, interest in the application of portable devices, specifically related to industrial inline use, has led to several studies comparing benchtop and portable devices, as well as investigations into data modeling and the development of prediction models [16].

NIR spectroscopy (NIRS) has demonstrated high sensitivity to changes in membrane structure, mainly due to its ability to detect characteristic vibrational frequencies associated with each functional group. Considering that bacterial membrane structures comprise a combination of macromolecules, including proteins, polysaccharides, nucleic acids, and lipids, the NIR spectrum of each bacterium exhibits a distinctly specific absorption signature linked to its genetic information [15,17].

NIRS can be combined with aquaphotomics, which provides further information regarding the samples by revealing sensitive physico-chemical changes through the comparison of water spectral patterns. These patterns reflect the interactions between water molecules and their surrounding environment, an approach related to the formation and breakdown of component bonds, driven by biological and chemical changes. Studies related to this “water molecular mirror” concept have already contributed scientifically to the field of microbiology by classifying bacteria strains based on probiotic strength, monitoring fermentation processes in real time, and evaluating the viability of commercial probiotic supplements. However, in the field of functional foods, in which consumer awareness is becoming more prominent, the association between fruit juice media and probiotics requires further investigation, which brings the combined NIR and aquaphotomics approach as a modern alternative for enhancing safety, quality control, and fermentation process optimization [17,18,19,20,21,22].

The aim of our research was to determine the applicability of NIRS and aquaphotomics for the characterization of different bacterial species in fruit juice media, including two probiotic strains as monoculture (*Bifidobacterium longum* and *Lactobacillus salivarius*) and one mixed-culture sample (containing both *Bifidobacterium longum and Lactobacillus salivarius*). A further goal was to develop models for the data-driven control of the fermentation process, enabling rapid prediction of key parameters such as pH and cell count based on media interactions through the chemical bond changes and consequently their spectral absorption.

## 2. Materials and Methods

### 2.1. Materials and Media for Culture

Fruit juices, 25% Hey-Ho plum juice (ELMA, Ersekhalmi, Hungary), 50% Happy Day sour cherry nectar (Rauch Hungary, Budapest, Hungary), and 100% Solevita apple juice (Lidl, Neckarsulm, Germany), were purchased from the local market. All chemicals were analytical-grade and were from Sigma-Aldrich (St. Louis, MO, USA), VWR International Kft. (Debrecen, Hungary).

De Man, Rogosa, and Sharpe (MRS) broth contained (per liter) 10g of proteose peptone, 4 g of yeast extract, 8 g of meat extract, 20 g of glucose, 5 g of sodium acetate, 2 g of tri-ammonium citrate, 2 g of K_2_HPO_4_, 0.2 g of MgSO_4_, 0.05 g of MnSO_4_, and 1 mL of Tween80.

Trypticase–phytone–yeast extract (TPY) broth contained (per liter) 10 g of trypticase peptone, 5 g of phytone peptone, 5 g of glucose, 2.5g of yeast extract, 1 mL of Tween80, 0.5g of cysteine-HCl, 2 g of K_2_HPO_4_, 0.5 g of MgCl_2_ ·6H_2_O, 0.25 g of ZnSO_4_ · H_2_O, 0.15 g of CaCl_2_, and 0.03 g of FeCl_3_ 6H_2_O. The culture media were sterilized at 121 °C for 15 min.

### 2.2. Microorganisms

Two probiotic bacteria, namely the *Bifidobacterium longum* DSM 16603 strain (BL) and the *Lactobacillus salivarius* HA-118 strain (LS), were purchased from Probiotical S.p.A (Novara, Italy) and Lallemand Health Solutions (Quebec, QC, Canada), respectively. The bacteria were propagated in the TPY or MRS broth before use.

### 2.3. Preparation of Probiotic Fruit Juices

The juice blend was made using commercially available fruit juices, such as 25% plum juice, 50% sour cherry nectar, and 100% apple juice, mixed in a ratio of 1:1:1. The pH of the blended juice was adjusted to a pH of around 6.5 by 4 N sterile NaOH.

The fermentation was initiated by adding 5% *Bifidobacterium longum* and *Lactobacillus salivarius* monocultures (cc. 10^7^ CFU/mL in fruit juice) grown in advance in TPY and MRS broths, respectively, for 24 h, or in a total of 5% of mixed cultures of the above probiotic strains. In the case of mixed-culture fermentation, a 1:1 ratio of the strains was applied. In the case of *Lactobacillus salivarius*, the fermentation was conducted in an incubator at 37 °C for 24 h, while in the cases of *Bifidobacterium longum* and mixed culture, the juices were placed in an anaerobic jar gas-pack system at 37 °C for 24 h. Sampling was performed at the 0, 4, 8, 16, and 24 h of fermentation in three replicates. The samples were kept frozen until analyses.

### 2.4. Applied Methods

#### 2.4.1. Determination of pH

The pH of the sampled juices was measured by a Mettler Toledo SevenMultiTM device (Columbus, OH, USA). Before the measurements, the instrument was calibrated with the appropriate buffer solutions.

#### 2.4.2. Determination of Probiotic Cell Count

First, a serial ten-fold dilution was prepared from the juice samples. Subsequently, the properly diluted juice samples were pipetted into the Petri dishes and plates were pouredwith MRS agar and TPY medium for *Lactobacillus* and *Bifidobacterium*, respectively. The Petri dishes were incubated at 37 °C for 48–72 h. After incubation, the grown colonies were counted and expressed in CFU/mL.

#### 2.4.3. Acquisition of the Spectra

For non-destructive spectral analysis of fermentation, the prepared probiotic fruit juices were subjected to near-infrared scanning using the Rapid Liquid Analyzer (RLA) module of XDS benchtop spectrometer (Metrohm, Herisau, Switzerland) in a transmission measurement setup. The spectral data were collected in the wavelength range of 400–2500 nm using a glass cuvette with a path length of 1 mm. The scanning was performed in a randomized order with three consecutive scans. During the measurement, the samples’ temperature was maintained at 35.0 ± 0.1 °C. Between each sample, the cuvette was emptied, rinsed three times with distilled water, drained, and then rinsed three times with the next sample. The cuvette-filled sample was then also tempered for 60 s before spectral recording.

A total of 117 spectra [13 samples × 3 replicates × 3 cons. scans] were recorded and later analyzed.

#### 2.4.4. Statistical Analyses

##### Evaluation of pH and Cell Count

The pH and cell count results of fruit juices with probiotic bacteria were evaluated with univariate data analysis. Descriptive statistics and box plot analysis were used for preliminary characterization of the analyzed samples. First, a two-way analysis of variance (ANOVA) of both pH and cell count was conducted to determine significant different characteristics among bacterial strains, fermentation times, and their respective interactions (bacterial strain * fermentation time). Subsequently, one-way ANOVA, followed by Tukey’s HSD pairwise comparison (*p* < 0.05 significance level), was performed, with ANOVA tests first performed within each microbial strain to detect the significant effect of fermentation time. Then, modeling was performed on data filtered by fermentation time to detect the effect of the microbial strain itself on pH and cell count. Significant differences were indicated by different lower-case letters in the former case, and by different capital letters in the latter case on the box plots [23,24].

##### Evaluation of the Spectral Data

Multivariate data analysis was performed on the NIR spectroscopic results. The NIR spectra were evaluated in the wavelength range of 1300–1600 nm using R-project v 4.1.1 (2021) and the aquap2 package [25].

Data evaluation started with the inspection of the raw and difference spectra, the latter calculated by subtracting the averaged spectrum at 0 h of fermentation from the spectra recorded at predetermined time intervals of fermentation for each culture separately. Various spectral pre-processing treatments were applied before further modeling. Smoothing was performed with the Savitzky–Golay filter using second-order polynomial and various points. This was followed by one of the following transformations: detrending (elimination of polynomial baseline tendencies), MSC (baseline shift reduction), standard normal variate (SNV), first derivative (removal of constant offsets), or second derivative (removal of linear offsets) with different data point frames. The article summarizes the best models obtained with different pretreatments.

PCA was applied to map spectral patterns in the data, while PCA-LDA in combination was performed to detect the effect of the progression of fermentation time. The optimal number of principal components (NrPCs) was determined with three-fold cross-validation, ensuring that consecutive scans of the same samples were always treated together. This means that these scans were either included in the training set or the validation set but never distributed between the two. An algorithm collected and compared LDA training and validation accuracies up to the predefined 30 NrPCs. The NrPCs providing the smallest difference between the accuracies of model building and cross-validation, as well as the highest validation accuracy, were used to build the optimized model. Modeling was also performed with three-fold cross-validation, but with the approach of always omitting spectral data by the sample group and then projecting in the model according to the three-measurement repetition. This was repeated three times in total so that all data were included at least once during model construction and validation, again ensuring that consecutive scans of the same samples were always treated together.

Partial least squares regression (PLSR) was applied to predict pH and cell count based on the pretreated spectral data. The predictive models were tested similarly to the PCA-LDA models, by three-fold measurement cross-validation. The model fitting accuracy of the PLSR was given by the coefficient of determination (R2) and the root mean square error (RMSE) during calibration (C) and validation (CV). The R-based algorithm tested the number of latent variables (NrLVs) optimal for model construction and selected the one with minimal RMSE values. Given the high biological variation, i.e., low repeatability of cell count determination among replicates, the preliminary evaluations showed that spectra averaged by fermentation time resulted in more reliable models, so PLSR modeling was performed on averaged data in the case of cell count determination.

##### Evaluation of the Aquagrams

Previous studies presented by Tsenkova et al., have introduced 12 characteristic wavelength ranges which summarize specific water bands for the first overtone region of water (1300–1600 nm), called water matrix coordinates (WAMACs), which represent differences in water molecule structures along the radial axes.

The aquaphotomics’ radar plot-like difference aquagrams were employed to illustrate the spectrally identifiable changes observed in the water absorbance spectral patterns (WASPs), which occur in fermenting juices with high water content. The aquagrams show the normalized results calculated on specific water matrix coordinates proposed by Tsenkova in 2009 [26,27]. The information is presented by means of average normalized absorbance values from the collection of spectra, using R-project v 4.1.1 (2021) and a package [25].

## 3. Results and Discussion

### 3.1. Changes in the Quality Characteristics of the Probiotic Fruit Juices

**Figure 1:**
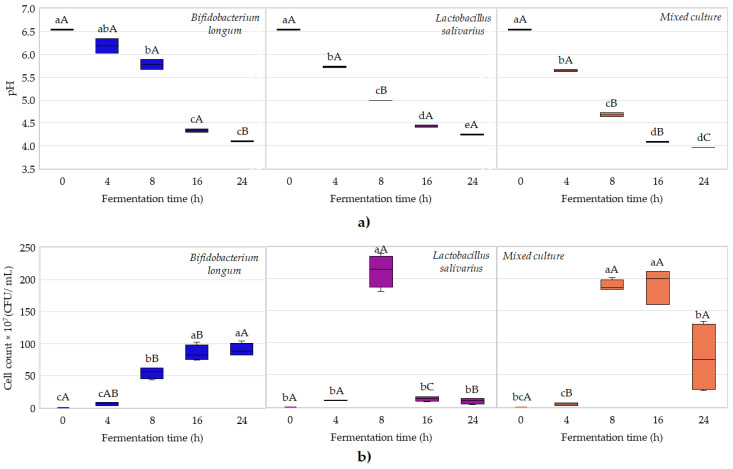
Box plot diagram of changes in reference properties during the probiotic fermentation of fruit juices. Changes in pH value by starter culture (**a**); changes in microbial cell count by starter culture in the juices (**b**). In the box plots, lower-case letters indicate significant differences by fermentation time, while capital letters indicate significant differences by probiotic culture.

During the fermentation process of blended fruit juices, changes in pH and cell count were monitored, and the results are shown in Figure 1.

Based on the results shown in Figure 1a, the pH values of all cases decreased from pH 6.5 to approximately pH 4.0 after 24 h of fermentation. The two-way ANOVA results demonstrated significant differences among bacterial strains, fermentation times, and their interactions. In all cases, the values of p were highly significant (*p* < 0.0001). The smallest differences were obtained for fermentation times between 16 and 24 h for all the three groups. Based on one-way ANOVA results, in the case of *Lactobacillus salivarius* HA-118, the pH values decreased significantly between sampling points, while in the cases of *Bifidobacterium longum* DSM 16603 and the mixed culture, the pH values decreased significantly until 16 h of fermentation, and after that, they were close to stationary. These results are in agreement with ones reported by Havas et al., (2015) as well as by Tran et al., (2020) [28,29].

Considering bacterial activity, a decrease in pH values is evident, since short-chain fatty acids such as lactic acid, propionic acid, succinic acid, and acetic acid are produced because of the fermentation process. The production of both acetic acid and lactic acid during sugar fermentation is also attributed to heterofermentative lactic acid bacteria, such as *Bifidobacterium*. Suido et al. (2008) also reported in their study that fermented broccoli with *Bifidobacterium* contained higher amounts of acetic acid (63 mM) compared to the amount of lactic acid (26 mM), while fermented broccoli with *Lactobacillus pentosus* did not have any acetic acid in the end-product after fermentation. In addition, some *Lactobacillus salivarius* strains have been shown to contribute to improving probiotic quality due to their high production of exopolysaccharide, which is a secondary metabolite mainly produced under unfavorable environmental conditions to protect the microorganisms [6,30].

Regarding cell growth, the different strains and their mixture showed very different, presumably strain-specific reproductive patterns in the juices. The growth of *Bifidobacterium longum* followed the typical “habitual” bacterial growth kinetics, with a prolonged lag and exponential phase, extending into a stationary phase (Figure 1b). The decline phase with cell death had not yet occurred. In the case of the *Lactobacillus salivarius* strain, an extremely high microbial count (2 × 10^9^ CFU/mL) was reached at 8 h of fermentation and then decreased drastically in the second half of the fermentation. This behavior was also reported by Mourad et al., (2025) when they performed the fermentation of the white egg white-drink (ToTu^TM^) [31]. Among others, this may be related to the pH sensitivity of the cells or substrate deficiency due to the high microbial count. In contrast to pH, the microbial count clearly shows the apparently complementary interaction between the two strains. In the case of the mixed culture, a prolonged lag followed by a rapid log phase and then a decline phase in the last 8 h of fermentation was observable. The two-way ANOVA results demonstrated significant differences among bacterial strains, fermentation times, and their interactions. In all cases, the values of p were highly significant (*p* < 0.0001). Significant differences between sample groups became more pronounced as fermentation progressed.

The type of media, their content, such as phenolic compounds, and pH, are factors with the greatest impact on probiotic cell survival. For example, phenolic compounds have shown antimicrobial activities against a variety of microorganisms, including lactobacilli, in a previous study comparing cranberry to pomegranate juice [13]. This could be one of the reasons why the Lactobacilli cell count decreases faster than the others. As a result of the application or the development of new media environments in the functional foods sector, some techniques such as encapsulation are being applied to improve the survival of probiotic strains [13].

### 3.2. Results of NIR Spectroscopy Analysis of the Probiotic Fruit Juices

#### 3.2.1. Preliminary Inspection of the NIR Spectra of the Probiotic Fruit Juices

**Figure 2:**
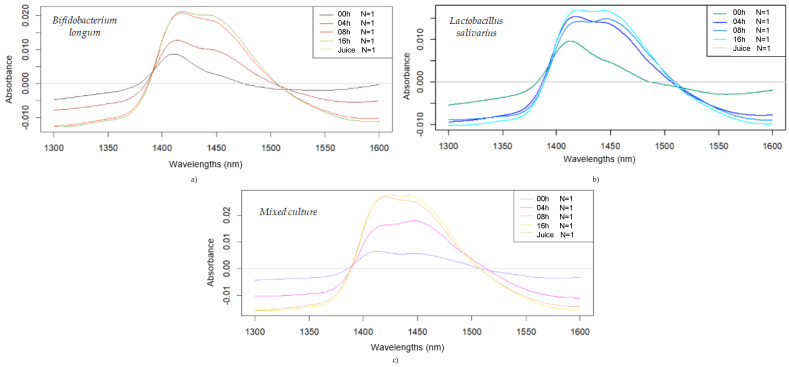
Smoothed (2nd-order polynomial, 35 points), detrended, and averaged difference spectra obtained during fermentation of fruit juices: difference spectra of *Bifidobacterium longum*-fermented juices (**a**); *Lactobacillus salivarius*-fermented juices (**b**); and mixed culture-fermented juices (**c**).

The difference spectra in the wavelength range of 1300–1600 nm are presented in Figure 2 to compare the influence of probiotic fermentation for each bacterial strain. The difference spectra, in general, for all strains, reveal increasing absorbance as fermentation progressed, reaching the maximum in the region of 1400–1450 nm. Regarding the intensity of absorbance, *Bifidobacterium longum* presents slightly higher values compared to the difference spectra of *Lactobacillus salivarius*. In addition, the difference spectra for mixed cultures have a similar absorbance intensity to *Bifidobacterium longum*. The spectral change follows a logical trend with the fermentation in all three cultures, but differences in the timing of the change can be observed. In the case of *Bifidobacterium longum* and the mixed culture, a more equal distribution of the subtracted spectra could be noticed at the 24 h fermentation time, while the spectral pattern of *Lactobacillus salivarius* presents a more similar pattern after 4 h of fermentation. It can also be noticed that the ratio of the two main bands around 1450 nm, representing the ratio between the less hydrogen-bonded and more hydrogen-bonded water structures, changes differently for the different fermentation times and bacteria cultures.

Some light is physically scattered, reflecting the increasing turbidity due to the higher microbial density as fermentation progresses [32], while chemical changes within the base juice are also reflected through spectral changes. The application of aquaphotomics has already been validated for evaluating different interactions within the medium resulting from bioactivity, as well as for assessing polysaccharides and their solubility in water [33].

It is known that absorption and scattering properties change during the fermentation process, in which the bacterial growth, stationary, and decline phases result in changes in optical properties.

#### 3.2.2. Principal Component Analysis of the NIR Spectra of Probiotic Fruit Juices

**Figure 3:**
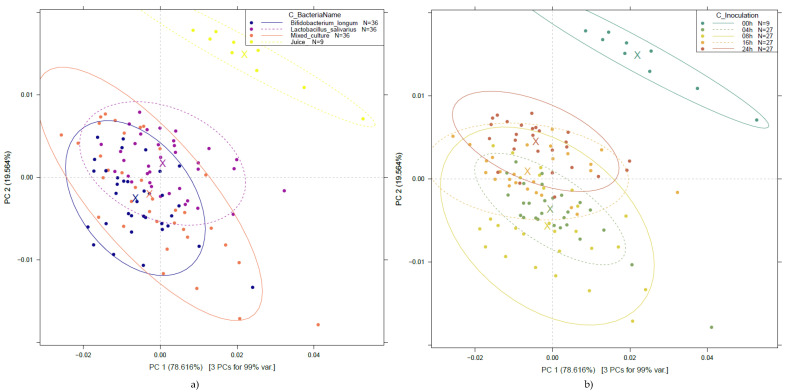
PCA of probiotic fruit juices for all the three bacterial cultures across various fermentation periods: PCA score plot by bacterial strain (**a**); by fermentation time (**b**). Ellipses represent 95% confidence intervals (Savitzky–Golay smoothing with 43 points and detrending, n = 117).

To investigate the effect of probiotic strains and fermentation on fruit juices, PCA was performed on the entire pretreated dataset. The results, presented in Figure 3, show that the first two PCs (PC 1 and PC 2) describe more than 98% of the variance present in the spectral data. The PCA score plots demonstrate clear separation for the unfermented fruit juice samples from the other groups, regardless of the type of bacterial culture (Figure 3a) and fermentation time (Figure 3b).

**Figure 4:**
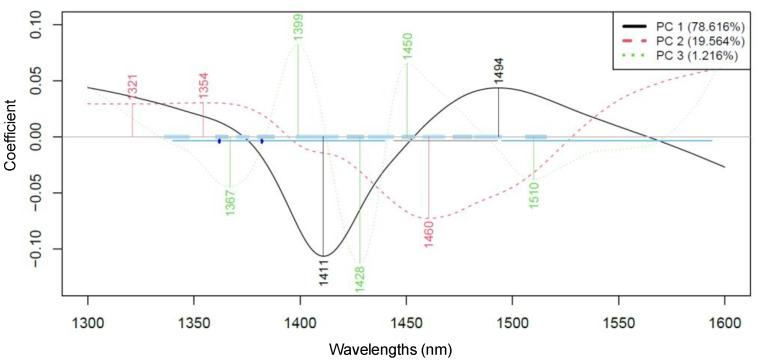
The wavelengths contributing most to the PCA of probiotic fruit juices for all three bacterial cultures across various fermentation periods (Savitzky–Golay smoothing with 43 points and detrending, n = 117).

As a partial result of the PCA, the wavelengths, i.e., loadings, that contributed most to each principal component were also obtained. Figure 4 shows the most influential wavelengths in PCA, which, for PC 1 and PC 2, were found to be at 1411, 1494 nm and 1460 nm, respectively.

The wavelength range associated with the first overtone region of water mainly refers to biological and aqueous systems, particularly involving intermolecular hydrogen bonds (C-H, O-H, and N-H). This assumption is proven by evaluating the medium composition and knowing that carbohydrates are one of the main components in fruit juices, together with water and phenolic compounds. Carbohydrates, in general, may have a free OH stretch absorption near 1440 nm, as also reported in crystalline sucrose, while phenolic compounds show absorption in the same region as aliphatic alcohols, with first overtones near 1400–1440 nm [26,27].

**Figure 5:**
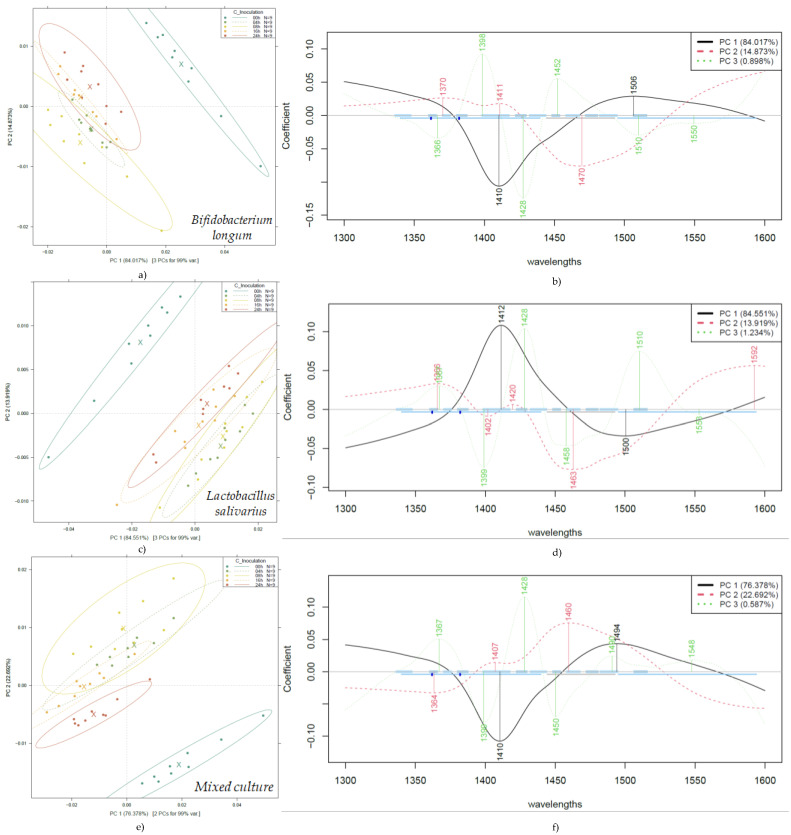
PCA according to fermentation time on probiotic fruit juices by bacterial cultures: PCA score plot of *Bifidobacterium longum*-fermented juices (**a**); PCA loading plot of *Bifidobacterium longum* (**b**); PCA score plot of *Lactobacillus salivarius*-fermented juices (**c**); PCA loading plot of *Lactobacillus salivarius* (**d**); PCA score plot of mixed culture-fermented juices (**e**); PCA loading plot of mixed culture (**f**). Ellipses represent 95% confidence intervals (n = 45 for each plot).

As a clear trend of separation could not be observed either among the groups of the different bacterial cultures or among the groups of different fermentation times in the above-detailed PCA results, modeling was also carried out on data filtered by strain, as presented in Figure 5.

Figure 5 illustrates a very clear separation of the unfermented fruit juice, present in the three evaluations. In contrast to Figure 3, by evaluating the PCA results, a separation according to the different fermentation times shows improved separation. For example, the mixed culture, after 24 h of fermentation, presents a clear distinction of the group in Figure 5e, and a similar behavior for *Bifidobacterium longum* is presented in Figure 5a. At the same time, Figure 5c shows overlapping information between 4–8 h and 16–24 h of fermentation.

The wavelengths contributing most to the separation according to fermentation times are 1410–1412 nm and 1494–1506 nm for PC 1, and 1364–1370 nm, 1407–1420 nm, and 1494–1506 nm for PC2, showing a very similar pattern of contributions across the three sample groups. In the case of *Lactobacillus salivarius*, there is also a contribution at 1592 nm for PC 2, which can be considered a distinguishing feature in comparison to the other groups.

#### 3.2.3. Linear Discriminant Analysis of the NIR Spectra of the Probiotic Fruit Juices

**Figure 6:**
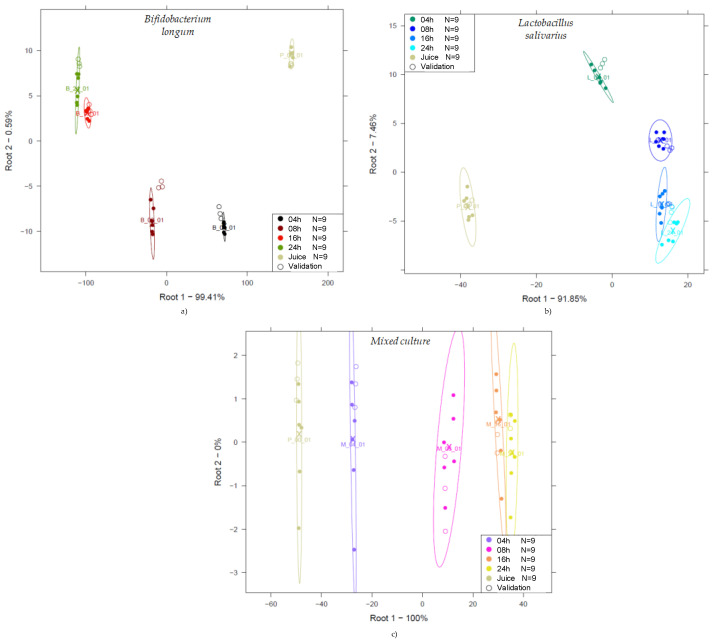
PCA-LDA classification according to fermentation time (0, 4, 8, 16, and 24 h) of probiotic juices: LDA score plot of *Bifidobacterium longum*-fermented juices (Savitzky–Golay smoothing with 21 points, n = 45, NrPCs = 4) (**a**); LDA score plot of *Lactobacillus salivarius*-fermented juices (detrending, n = 45, NrPCs = 6) (**b**); LDA score plot of mixed culture-fermented juices (msc, n = 45, NrPCs = 2) (**c**).

PCA-based LDA was performed to classify probiotic fruit juice spectra according to the degree of fermentation. To explore exactly what the detectable effects of each strain are, analyses were conducted on a strain-by-strain basis. Figure 6 presents the resulting LDA score plots, depicting evident discrimination among the sample groups. Model building and testing accuracy were 100% in all cases.

The unfermented juices (indicated as Juice in Figure 6) are clearly distinct from the data points representing the other groups. For monoculture fermentations, the data points are typically arranged in a kind of U shape, while for the mixed culture, the data are arranged along the first discriminant variable. It is typical that towards the end of fermentation, the 16 and 24 h results are positioned close to each other, especially for the *Lactobacillus salivarius* group.

A possible explanation for the fact that groups corresponding to the fermentation times of 16 and 24 h are close to each other, showing overlapping in some cases, is that as the fermentation time increases, the number of the cells in the sample also increases. As a result, it will affect the multiplicative scattering of light during the acquisition of the spectra via transmission arrangement depending on the growth cycle of cells, impacting the acquired absorbance spectra and consequently the collected data.

In the present study, pH and cell count are important parameters for tracking the fermentation process, and both indicators are relevant to the viability of probiotic fruit juices with regard to their further applicability. For such samples, non-destructive estimation of these traits can be challenging. PLS regression was used to determine the accuracy with which changes in pH and cell count during fermentation could be predicted using a combination of NIR spectroscopy and aquaphotomics. To describe the accuracy observed for each starter culture, the analysis was performed separately for each strain.

**Figure 7:**
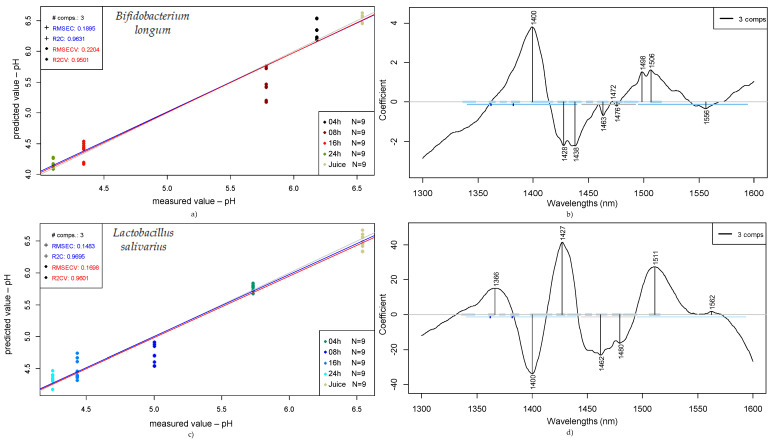
Prediction of pH of fruit juices according to fermentation time (0, 4, 8, 16, and 24 h): PLSR scatter plot of *Bifidobacterium longum* samples (detrending, n = 45, NrPCs = 3) (**a**); PLS regression vectors for *Bifidobacterium longum* (**b**); PLSR scatter plot of *Lactobacillus salivarius* (SNV, n = 45, NrPCs = 3) (**c**); PLS regression vectors for *Lactobacillus salivarius* (**d**).

The results obtained for the pH value prediction with PLSR are presented in Figure 7 for each one of the single strains—*Bifidobacterium longum* and *Lactobacillus salivarius*.

From the data obtained for pH prediction through partial least squares regression (PLSR) presented in Figure 7, *Bifidobacterium longum* samples show a coefficient of determination for calibration (R2C) of 0.96 with a root mean square error of calibration (RMSEC) of 0.19. In the same figure, the stated values after cross-validation were 0.95 for the R2CV and 0.22 for the RMSECV. On the other hand, for the *Lactobacillus salivarius* samples, an R2C of 0.97 was obtained, with a root mean square error of calibration of 0.15, while for the cross-validation, R2CV was 0.96 and RMSECV was 0.17.

In this case, the wavelengths contributing most for *Bifidobacterium longum* were 1400, 1428–1438, and 1498–1506 nm, while for the *Lactobacillus salivarius*, they were 1366, 1400, 1427, 1462–1480, and 1511 nm.

Comparing the presented coefficients for the single strains (*Bifidobacterium longum* and *Lactobacillus salivarius*), regarding pH prediction, as shown in Figure 7, both strains present similar values and a clear tendency, in which the pH decreases and the fermentation time increases—an expected behavior of lactic acid bacteria during the progression of the fermentation process.

The wavelengths contributing most to the pH prediction according to PLSR show some very distinct coefficients according to the microorganism; for example, 1366 nm is characteristic of *Lactobacillus salivarius*. In addition, some coefficients can be related to both microorganisms, such as 1400, 1427–1428, and 1506–1511 nm, representing the main contributors for the predicted models, and they also impact the mixed culture.

Considering that during the fermentation process, both lactic acid (or 2-hydroxypropanoic acid) and acetic acid are produced by the LAB present in the medium, we can expect that these two products will be represented on the NIR spectra collected for the samples.

For acetic acid, the second combination region for CH (1300–1600 nm) shows two representative peaks around 1350 nm and 1370 nm and an increase in the absorbance (higher than 0.5) after 1500 nm. In comparison, lactic acid or 2-hydroxypropanoic acid, which is also present after the fermentation process, shows characteristic wavelengths in the range between 1300 and 1600 nm, represented by 1333, 1429, and 1538 nm (values obtained from an absorbance spectrum related to propanoic acid).

**Figure 8:**
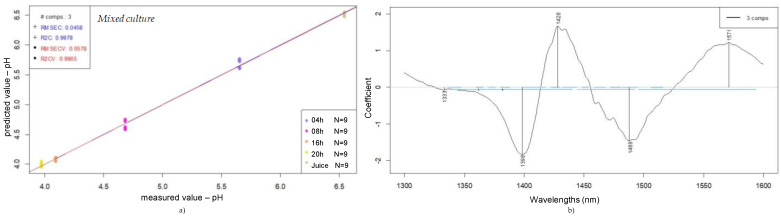
Prediction of pH of fruit juices according to fermentation time (0, 4, 8, 16, and 24 h): PLSR scatter plot of mixed-culture samples (SNV, n = 45, NrPCs = 3) (**a**); PLS regression vectors for mixed culture (**b**).

For the purpose of comparing the relationship between the utilized bacteria type and the wavelengths contributing most, the prediction model for the pH value of a mixed culture is presented in Figure 8.

For the medium containing both LAB strains, the behavior observed includes the common wavelength range previously established—1398, 1428, and 1488 nm—and also features a specific wavelength at 1571 nm. The mixed-culture medium represents not only the interactions within the juice medium itself, but also, equally importantly, the relationship between the strains present in the sample and their chemical and microbiological interactions.

**Figure 9:**
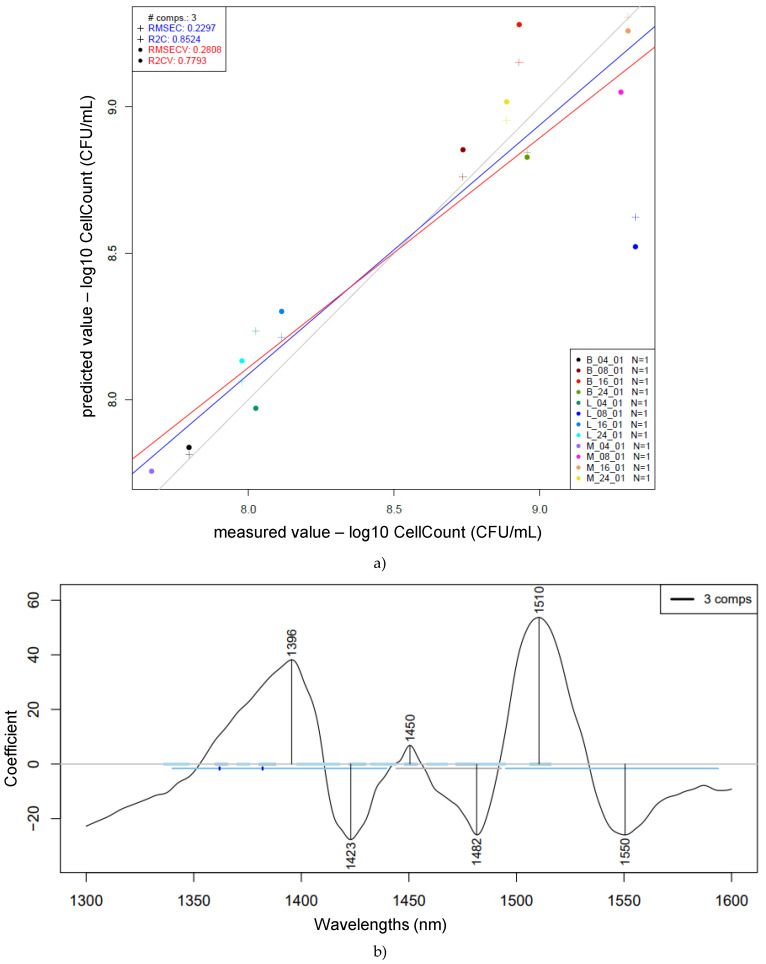
Prediction of log10 cell count (CFU/mL) based on the whole pretreated (Savitzky–Golay smoothing, 2nd-order polynomial, 21 points, msc) spectra of single and mixed strains: PLSR scatter plot of cell count prediction (n = 12, NrPCs = 3) (**a**); PLS regression vector of cell count prediction (**b**). The figure legend indicates the group name based on bacterial strain (BL = *Bifidobacterium longum*, LS = *Lactobacillus salivarius*, and MC = mixed culture) present in the sample and fermentation time (4, 8, 16, 24)—e.g., LS_16h: *Lactobacillus salivarius*, 16 h of fermentation.

The best prediction model presented in Figure 9, shows a coefficient of determination for calibration (R2C) of 0.998 with a root mean square error of calibration (RMSEC) of 0.05. In the same figure, the stated values after cross-validation were 0.996 for the R2CV and 0.06 for the RMSECV.

Comparing the three prediction models developed for pH, the one presented for the mixed culture has shown better coefficients of determination and root mean square errors, for both calibration and cross-validation, in comparison to the single ones. These results support that NIR is an efficient method in predicting the pH during the fermentation tracking of probiotic fruit juices.

The advantage of introducing NIR as a tool for microbiological process control lies in its ability to offer an alternative for determining the optimal stage of probiotic activity by associating pH and cell count. In addition, it is a non-destructive and fast correlative method.

In addition, the compiled data obtained for log cell count prediction (CFU/mL) according to PLSR are presented in Figure 9 and consist of the mean square error and the R-squared values calculated by cross-validation for the averaged samples (n = 12). The prediction model was established based on the average for each one of the groups, separated by sample type and the corresponding fermentation times. The reason for using averaged values is the microbiological behavior observed during the bacterial growth curve.

The best prediction model obtained provided the following wavelengths with the highest loadings: 1396, 1423, 1450, 1482, 1510, and 1550 nm. A previous study related to predictive modeling of probiotic CFU counts established a model with wavelengths similar to the ones introduced in this study—namely, 1458 nm, which is associated with OH stretching, and 1484 nm, associated with OH/NH stretching [22].

In Figure 9, the stated coefficient of determination for calibration (R2C) was 0.85, with a root mean square error of calibration (RMSEC) of 0.23. The values obtained after cross-validation were an R2CV of 0.78 and an RMSECV of 0.28. In comparison to the previously presented prediction models for pH, there are similarities between the most influential wavelengths. The prediction model for log cell count (CFU/mL) parameter prediction shows 1396 nm, similar to the 1400 nm observed for pH prediction; 1423 nm, similar to 1427 and 1428 nm; 1482 nm, similar to 1488 nm (which was very characteristic of the mixed-culture samples); 1510 nm, similar to 1511 nm (which was very characteristic of *Lactobacillus salivarius*); and 1550 nm, aligning with the 1560–1571 nm range. Despite some variations in the predicted results, this study’s attempt to correlate NIR spectra with different fermentation times for single and mixed microorganism groups has presented good results, based on its coefficient of determination for calibration (R2C), cross-validation (R2CV), and the corresponding root mean square errors, considering the microbiological behavior of different microorganisms according to their specific growth rates.

#### 3.2.4. Aquagrams

**Figure 10:**
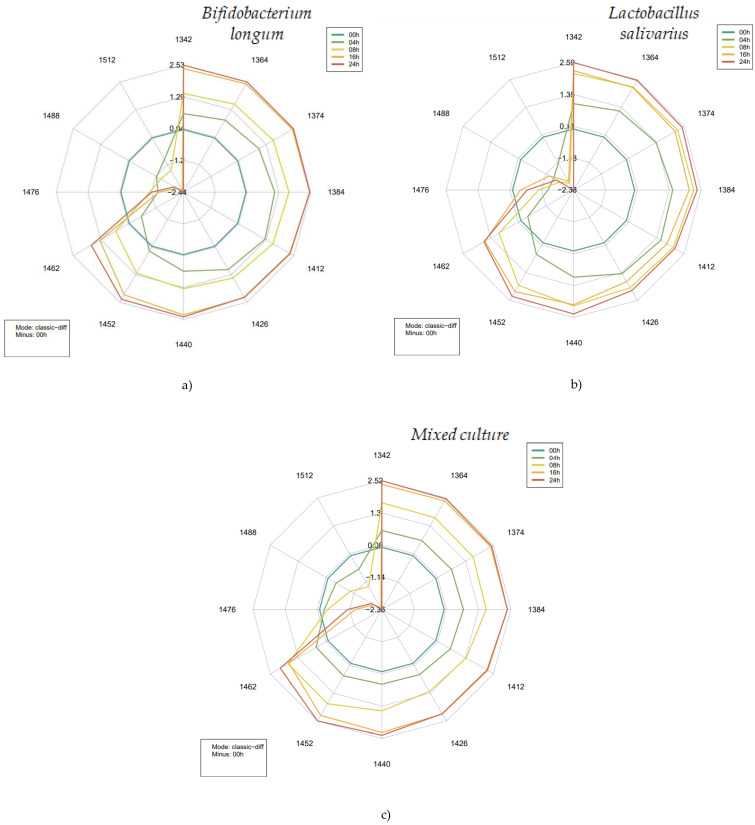
Aquagrams plots obtained according to the fermentation time (0, 4, 8, 16, and 24 h) for the undiluted samples of each single and combined bacteria strain type. (**a**) represents the obtained data for the *Bifidobacterium longum* group. (**b**) represents the obtained data for the *Lactobacillus salivarius* group. (**c**) represents the obtained data for the mixed-culture group.

To further investigate and pinpoint the changes during the probiotic fermentation of fruit juices and present these findings using a holistic approach, spectral pattern mapping was performed at 12 specific wavelengths (i.e., water matrix coordinates, WAMACs) consistently occurring in aquaphotomics-related research. The absorbance values at these WAMACs were used to calculate the aquagrams by probiotic culture. The aquagrams shown in Figure 10 detail the average spectral differences at certain times of fermentation compared to the results of the first measurement. These are visualized together with the water spectral patterns (WASPs), which could serve as integrative markers of probiotic fermentation in fruit juices.

The difference aquagrams clearly show characteristic patterns as the fermentation progressed. For all the three starter cultures, it was observed that the absorbance increased between 1342 and 1452 nm compared to the initial data, decreased around 1452–1462 nm, then increased monotonically, and decreased significantly at wavelengths above this range. In the range of 1462–1512 nm, a kind of balanced, gradual decrease in absorbance could be observed in the data obtained on samples inoculated with the two probiotic strains.

The activity of microbes leads to the formation or degradation of a number of components that molecularly bind water to different degrees or may affect the vibrational modes of water present in the juices. The aquagrams illustrate these metabolic processes well, revealing the relationship between the medium and its hydrogen-bonded and covalent water structures. Starting from the pure juice itself (0 h of fermentation), the number of the highly bonded water structures significantly decreases for the second half of the fermentation. Microbial activity intensifies the breakdown of substrates, namely larger organic molecules, like carbohydrates and peptides, which leads to the release of formerly molecularly bound water. As the level of probiotic activity increases, lactic acid and acetic acid (depending on the type of bacterial strain) are produced, which also leads to a higher availability of less hydrogen-bonded water [34]. Additionally, the release of volatile components should not be overlooked. All these phenomena can be observed in the range of 1342 to 1462 nm, following a clockwise motion.

Comparing the aquagrams to previous studies conducted in the field of probiotics proves that probiotic bacteria (species of *Lactobacillus* genus) show higher absorbance levels between 1365 and 1426 nm [21], in agreement with the data presented in Figure 10.

Taking into consideration *Bifidobacterium longum* and *Lactobacillus salivarius*, there is a constant decrease in the absorbance levels between 1426 and 1476 for 4 h of fermentation, while the mixed culture holds its absorbance intensity until 1462 nm, showing a slight decrease until 1488 nm and a sudden decrease towards 1512 nm.

The overlap between the fermentation times of 16 and 24 h is noticeable for both *B. longum* and the mixed culture, showing some differences in absorbance levels between 1440 and 1462 nm, showing similar behavior between the interactions. Although *L. salivarius* shows a very distinct behavior related to the fermentation times of 8, 16, and 24 h—there is an overlap between the times 8 and 16 h, and between 1364 and 1440 nm—the corresponding fermentation time of 16 h shows lower absorbance intensities in comparison to those of 16 and 24 h. At this point, we can highlight that the optimal absorbance value for the *Lactobacillus* was reached before the other groups, providing information on the best parameters (or wavelengths) for monitoring the fermentation process [21].

The relationship between the coordinates presented in Figure 10 and the corresponding water species has been discussed in many theoretical and empirical studies in the literature [17,20,26,34,35,36,37]. Absorption bands between 1336 and 1348 nm are mainly attributed to asymmetric stretching vibration of H_2_O–2*v_3_. Absorption in the ranges of 1360–1366 (C2), 1370–1376 (C3), and 1380–1388 nm (C4) reflects changes in the water solvation shell, protonated water clusters, and the water solvation shell. The increase in absorbance at 1364, 1374, and 1384 nm [38] marks water release or loss. Changes in absorbance at 1398–1418 nm (C5) and 1421–1430 nm (C6) can be associated with free or quasi-free water involved in hydration, directly linked to the decomposition of structural components present in the juices. Absorption at 1432–1444 nm (C7) reflects phase transitions in the system, mainly between liquid and vapor states, and may also be related to the release of water previously bound in, e.g., high-molecular-weight structures. The C8 and C12 WAMACs and above are associated with more and more bonded water and/or other molecular structures by hydrogen bonding [38,39]. Overall, the former can be explained by the fact that decomposition processes result in the release of an increasing number of previously bound small molecules, which are simultaneously hydrated and contribute to increased microbial activity, and consequently, turbidity.

## 4. Conclusions

The present study has proven that the region between 1300 and 1600 nm shows different characteristics, according to its spectra and absorbance values, implying a distinct absorption signature. This characteristic absorption signature is obtained by detecting vibrational frequencies through the collection of spectra, which can be associated with the tracking of the fermentation process for the probiotic fruit juice matrix.

The characteristic wavelengths impacting the prediction of the parameters, as well as the discrimination between the different microorganism groups (*Bifidobacterium longum* DSM 16603, *Lactobacillus salivarius* HA-118 and mixed culture) and fermentation times (4, 8, 16, and 24 h), have shown clear consistency by yielding similar contribution wavelength ranges during the data evaluation performed.

In this context, NIR, as a tool for tracking the fermentation process, allows us to comprehend the microbiological behavior of a specific strain in relation to the medium. In this way, a controlled environment would result in an optimized process for specific probiotic activity, defining an optimal point at which the probiotic strain reaches its maximum stress tolerance point, after which its activity starts to decrease.

The application of a multivariate data analysis creates robust prediction models, with results for pH showing coefficients of determination in the range of 0.96 to nearly 1 and root mean square errors of 0.05 and 0.19. On the other hand, for PLSR prediction of log cell count (CFU/mL), consistent with the expected optical changes in absorption and scattering, the validation model including single and mixed strains achieves a coefficient of determination of 0.85 and a root mean square error of 0.23.

Overall, NIR associated with aquaphotomics has proven good suitability as a tool for tracking different fermentation times, according to the different biochemical fingerprints of the samples, while probiotic activity is comprehended through the microbiological behavior of single and mixed microorganisms within the juice media.

In this way, it is possible to define an optimal point at which the probiotic strain reaches its maximum stress tolerance in a controlled environment, after which its activity starts to decrease, offering a promising application for process optimization and product quality improvement.

## Data Availability

The original contributions presented in the study are included in the article, further inquiries can be directed to the corresponding author.
